# Bi-Doped Pd Aerogels with Tensile-Strain-Induced Cascade Orbital Hybridization Boost H_2_O_2_ Selective Activation for Efficient Pesticide Distinction

**DOI:** 10.34133/research.1300

**Published:** 2026-06-05

**Authors:** Ruimin Li, Chengjie Chen, Lijun Hu, Dongbo Yan, Xiaotong Li, Xiangkun Jia, Yu Wu, Lei Jiao, Yanling Zhai, Chengzhou Zhu

**Affiliations:** ^1^ Institute of Molecular Metrology, College of Chemistry and Chemical Engineering, Qingdao University, Qingdao 266071, P. R. China.; ^2^ State Key Laboratory of Green Pesticide, International Joint Research Center for Intelligent Biosensing Technology and Health, College of Chemistry, Central China Normal University, Wuhan 430079, P. R. China.

## Abstract

The selective activation of H_2_O_2_ into hydroxyl radicals (•OH) through the synergistic interplay between the nanozyme’s internal orbital interactions and external orbital coupling with intermediates presents an important scientific challenge. In this study, we demonstrate that PdBi aerogels with controllable tensile strain first enhance the Pd d orbital energy level through p–d orbital hybridization, and subsequently, the hybridized Pd d orbitals couple sequentially with the molecular orbitals of H_2_O_2_, forming a cascade of orbital hybridizations. High-performance liquid chromatography quantitative analysis reveals that the engineered PdBi aerogels remarkably improve •OH selectivity from 73.3% to 95.4%, importantly boosting both the activation efficiency and selectivity. Experimental studies and theoretical calculations have demonstrated that the hybridization of the d orbitals of Pd and the p orbitals of Bi in PdBi aerogels initially raises the d-band energy level of the Pd atoms. During the activation of H_2_O_2_, the high-energy 4d_xz/yz_ and 4d_z_^2^ orbitals of Pd further hybridize with the 2π* and 5σ orbitals of H_2_O_2_, thereby enhancing the orbital interaction with H_2_O_2_ and creating optimal conditions for O–O bond cleavage and consequently enhancing the efficiency of •OH generation. Capitalizing on the differential reactivity patterns of various pesticides with PdBi aerogels, we develop a colorimetric sensor array for the discrimination and simultaneous detection of pesticide residues. Taking chlorpyrifos as an example, the detection limit is 0.23 μM, demonstrating good detection sensitivity. This work presents an orbital-level design strategy for creating highly selective nanozymes in H_2_O_2_ activation systems.

## Introduction

Hydrogen peroxide (H_2_O_2_) serves not only as an important reaction medium in environmental remediation and organic synthesis but also plays a pivotal role in biosensing and energy conversion [1–6]. However, the oxidizing capacity of H_2_O_2_ is inherently limited, necessitating efficient activation to convert it into reactive oxygen species (ROS) such as hydroxyl radicals (•OH), superoxide radicals (•O_2_^−^), and metal-oxo species (M=O) [7–9]. Among these, the •OH exhibits remarkable advantages in oxidizing organic compounds due to its extremely high redox potential and potent nonselective oxidizing capability, which can significantly amplify signals and thereby substantially enhance detection sensitivity [10,11]. Nevertheless, the spontaneous decomposition of H_2_O_2_ is inefficient. Its activation process heavily relies on catalyst performance. Among various strategies for activating H_2_O_2_, nanozymes, as a novel type of artificial catalyst, provide an effective approach to addressing the aforementioned issue due to their designable catalytic activity and controllable ROS-generating capacity. Therefore, the development of highly efficient nanozymes to achieve controllable •OH generation is crucial for improving both catalytic reaction efficiency and sensing performance.

To address these challenges, researchers have explored various regulation strategies, including interface engineering [12,13], dual-site engineering [14,15], and defect engineering [16,17]. While regulation strategies have improved H_2_O_2_ activation efficiency, precise control over the reaction pathway remains elusive [18]. The fundamental limitation originates from insufficient overlap between metal d orbitals and the σ orbitals of H_2_O_2_, leading to inefficient O–O bond cleavage [19–21]. Although electronic structure modulation and the d-band center theory have been widely used to enhance catalytic activity, there is still a lack of effective strategies that can achieve site-specific orbital hybridization [22]. Notably, existing studies have shown that d–d, p–d, and f–p–d orbital hybridization can importantly improve catalytic performance; however, these strategies often focus on the internal orbital interactions within the nanozyme and frequently overlook the subsequent hybridization process between the catalytically active sites and the target molecules [23,24]. In fact, it is this cascade orbital hybridization with the adsorbates that has a decisive impact on the activity and selectivity of catalytic reactions. Therefore, precisely triggering and regulating this crucial subsequent cascade orbital hybridization process remains a significant challenge in current research.

Herein, we propose an effective cascade orbital hybridization strategy using Bi-doped Pd (PdBi) aerogels. The Bi-induced tensile strain in the Pd lattice triggers pronounced p–d orbital hybridization, which in turn modulates the hybridization between the d orbitals of the catalytic sites and the σ orbitals of H_2_O_2_, thus forming a p–d–σ cascade orbital hybridization. This electronic manipulation importantly enhances the p–d–σ hybridization between the nanozyme and H_2_O_2_, promoting the selective activation of H_2_O_2_ and increasing the •OH selectivity from 73.3% to 95.4%. Experimental and theoretical analyses confirm that p–d hybridization elevates the d-band center of Pd, facilitating cascade orbital interactions with H_2_O_2_. This promotes O–O bond elongation (from 1.489 to 1.508 Å) and efficient •OH formation. Capitalizing on the differential inhibition of •OH generation by various pesticides, we design a colorimetric sensor array for the precise identification and simultaneous quantification of pesticide residues. This work not only advances the fundamental understanding of orbital-level nanozyme design but also provides a practical platform for environmental monitoring applications.

## Results and Discussion

### Characterization of the nanozymes

**Fig. 1.**
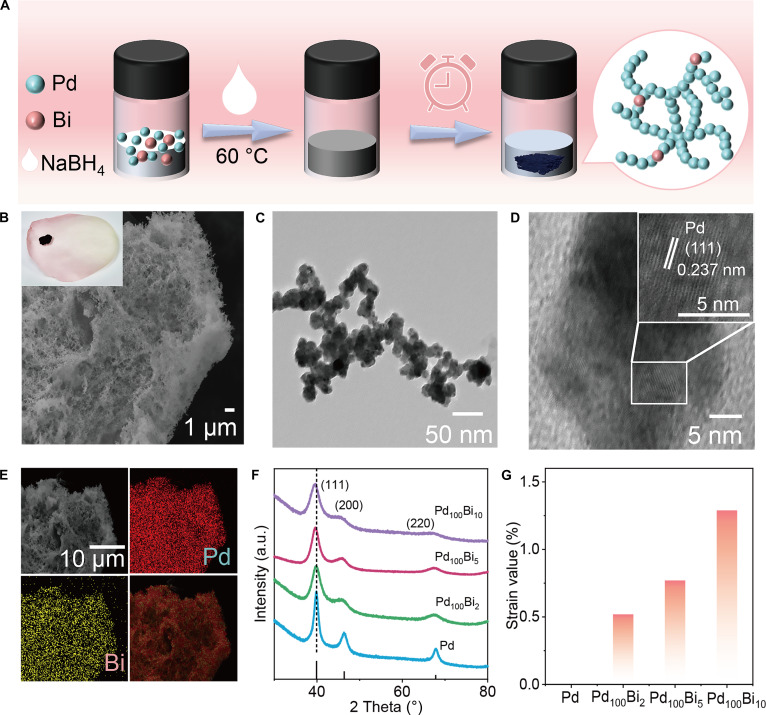
(A) Schematic illustration of the synthesis of Pd_100_Bi_5_ aerogels. (B) Scanning electron microscopy image (inset: digital photograph of Pd_100_Bi_5_ aerogel). (C) Transmission electron microscopy (TEM) image. (D) High-resolution TEM image. (E) Energy-dispersive x-ray spectroscopy mapping images of Pd_100_Bi_5_ aerogels. (F) X-ray diffraction patterns of Pd and PdBi aerogels. (G) Strain value of Pd and PdBi aerogels.

PdBi hydrogels were synthesized through the reduction of palladium acetylacetonate [Pd(acac)_2_] and bismuth (III) chloride (BiCl_3_) with sodium borohydride (Fig. 1A). The aerogels were obtained via a freeze-drying process of the as-prepared hydrogels (inset in Fig. 1B) [25]. Subsequently, inductively coupled plasma atomic emission spectrometry analysis indicated that the atomic ratio of Pd to Bi in the PdBi aerogels is 100:5 (defined as Pd_100_Bi_5_ aerogels). Additionally, pure Pd aerogels and Pd_100_Bi_x_ (x = 2, 10) were prepared for comparison. This composition is consistent with the molar ratio used in the synthesis process (Table S1). As shown in Fig. 1B and C and Fig. S1, the morphology of the resulting Pd aerogels and Pd_100_Bi_5_ aerogels is investigated by scanning electron microscopy and transmission electron microscopy (TEM), revealing 3D architectures composed of interconnected porous and extended nanowire networks [26]. The high-resolution TEM (HR-TEM) result demonstrates that the Pd_100_Bi_5_ aerogels have a lattice spacing of 0.237 nm ascribed to the (111) planes of face-centered cubic Pd aerogels (Fig. 1D). Besides, the selected area electron diffraction (SAED) also reveals the polycrystalline nature of Pd_100_Bi_5_ aerogels, which are indexed to (111), (200), (220), and (311) of Pd (Fig. S2). The uniformly distributed Pd and Bi elements in the Pd_100_Bi_5_ aerogels are further demonstrated by the energy-dispersive x-ray spectroscopy mapping images (Fig. 1E). The x-ray diffraction pattern of Pd and PdBi aerogels reveals the face-centered cubic polycrystalline structure, consistent with the SAED results (Fig. 1F). In contrast to Pd aerogels, the reflections of PdBi aerogels exhibit noticeable shifts toward lower 2*θ* values as the fraction of Bi increases, which suggests that doping Bi into the Pd lattice can produce tensile strain [27]. Quantitative analysis based on the Bragg formula shows that this tensile strain increases from 0.52% to 1.29% as the Bi content increases (Fig. 1G). Additionally, the (111) lattice stripe spacing is directly measured by HR-TEM, and it is found that the lattice spacing of Pd is 0.232 nm. As the Bi-doping amount increases, the lattice spacing gradually increases, confirming the existence of the strain (Fig. S3). Notably, the continuous increase in this tensile strain suggests that we can precisely control the electronic structure of the Pd lattice by adjusting the amount of Bi doping.

### Chemical state and electronic interaction

**Fig. 2.**
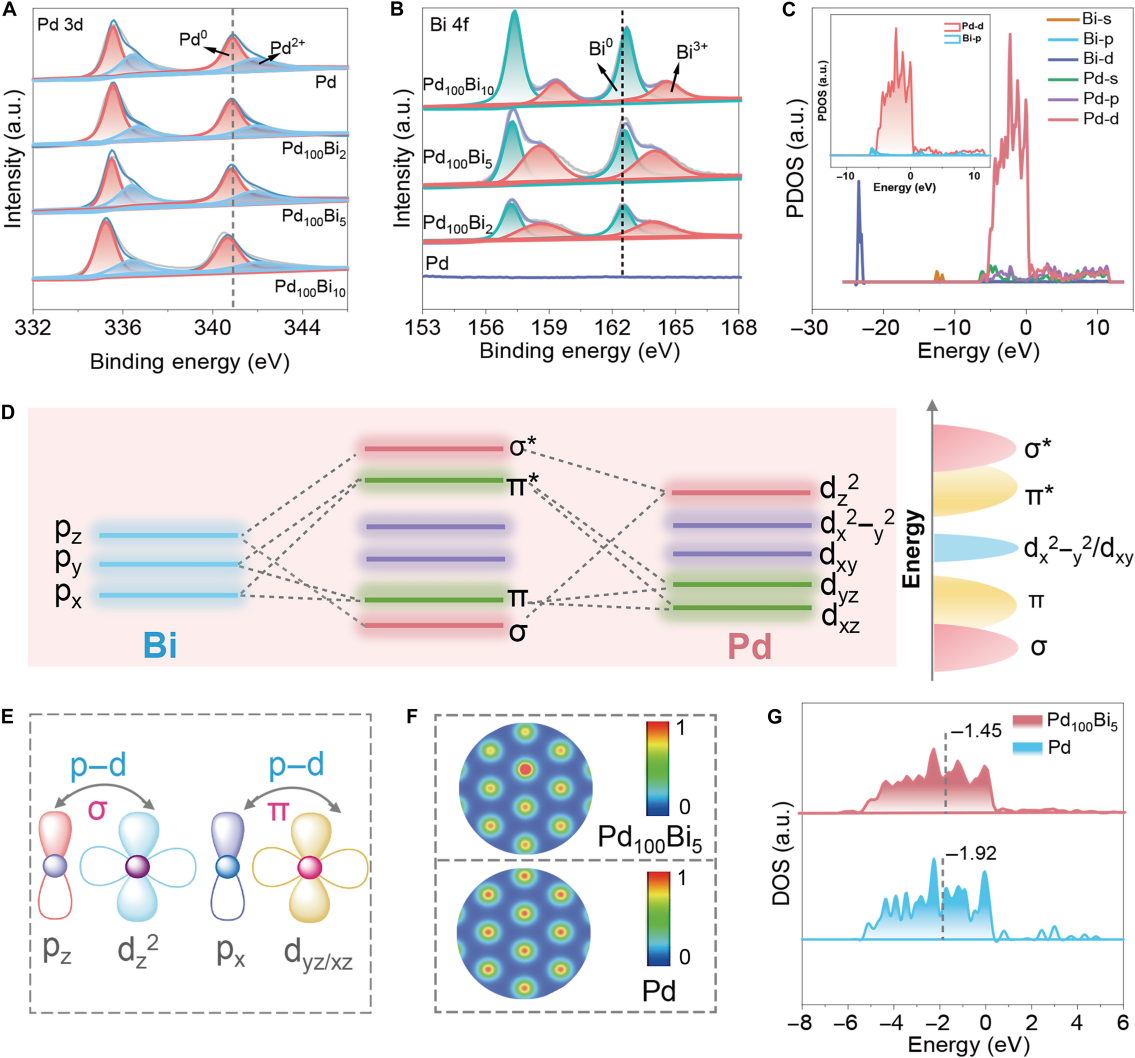
(A) Pd 3d and (B) Bi 4f x-ray photoelectron spectroscopy (XPS) spectra of Pd and PdBi aerogels. (C) Projected density of states (PDOS) of Pd_100_Bi_5_ aerogels (the inset depicts a plot of the Pd 4d orbital and Bi 6p orbital). (D) The p–d orbital hybridization distribution pattern and the energy level of the Pd–Bi orbital hybridized density of states of Pd_100_Bi_5_ aerogels. (E) Schematic illustration of the hybridization between the p orbitals of Bi and the d orbitals of Pd. (F) The charge difference on the surface of Pd and Bi for Pd_100_Bi_5_ aerogels and Pd aerogels. Red and blue colors represent the depletion and accumulation of electrons, respectively. (G) The calculated d-band center of surface Pd for Pd_100_Bi_5_ aerogels and Pd aerogels.

X-ray photoelectron spectroscopy (XPS) was performed to investigate the electronic structure and surface composition. With increasing Bi-doping concentration, a gradual decrease in the binding energy of Pd is observed, accompanied by a concurrent increase in the binding energy of Bi, indicating a clear electron transfer from Pd to Bi atoms (Fig. 2A). More importantly, in the Bi 4f spectra, 2 pairs of peaks are associated with Bi^0^ and Bi^3+^ species. The relative content of Bi^3+^ shows a trend of increasing first, then decreasing, with increasing doping amount. This trend is crucial for understanding the aforementioned charge transfer behavior and its influence on catalytic performance. The Bi^3+^ species possess empty p orbitals, and their increased content importantly enhances the p–d orbital hybridization between Pd and Bi, thereby modulating the electronic interactions within the nanozyme. The XPS results provide direct evidence for the modulation of electronic structure in PdBi aerogels (Fig. 2B).

To verify the presence of orbital interaction between Pd and Bi, the projected density of states (PDOS) was calculated (Fig. 2C). The peaks of the Pd 4d orbitals align well with those of the Bi 6p orbitals, indicating strong p–d orbital hybridization. Based on the orbital symmetry, the d_z_^2^ orbital of Pd 4d can directionally hybridize with the p_z_ orbital of Bi 6p to form σ and σ* orbitals. Meanwhile, the p_x/y_ orbitals of Bi 6p can directionally interact with the d_xz_/d_yz_ orbitals of Pd 4d, generating π and π* orbitals (Fig. 2D and Fig. S4). Additionally, due to the mismatch in orbital orientation with the Bi 6p orbital, the d_xy_ and d_x_^2^_−y_^2^ orbitals of Pd 4d are considered nonbonding. As shown in Fig. 2E and F, the p–d hybridization center on the orbitals leads to electron transfer from Bi to Pd, which can be verified through XPS spectra and differential charge density analysis. The crucial principle is that the lattice stretching strain caused by Bi doping (Fig. 1G) effectively promotes the occurrence of this p–d hybridization. With the assistance of the strain, the p–d hybridization between Pd and Bi not only induces charge transfer but also adjusts the symmetry and energy level distribution of the Pd d orbitals, thereby shifting the d-band center of Pd toward the Fermi level (Fig. 2G and Fig. S5). It is precisely this strain-induced p–d hybridization that directly regulates the local electronic structure of Pd, laying the foundation for the subsequent cascade orbital hybridization (p–d–σ) in the H_2_O_2_ activation process.

### Catalytic performance of nanozymes

**Fig. 3.**
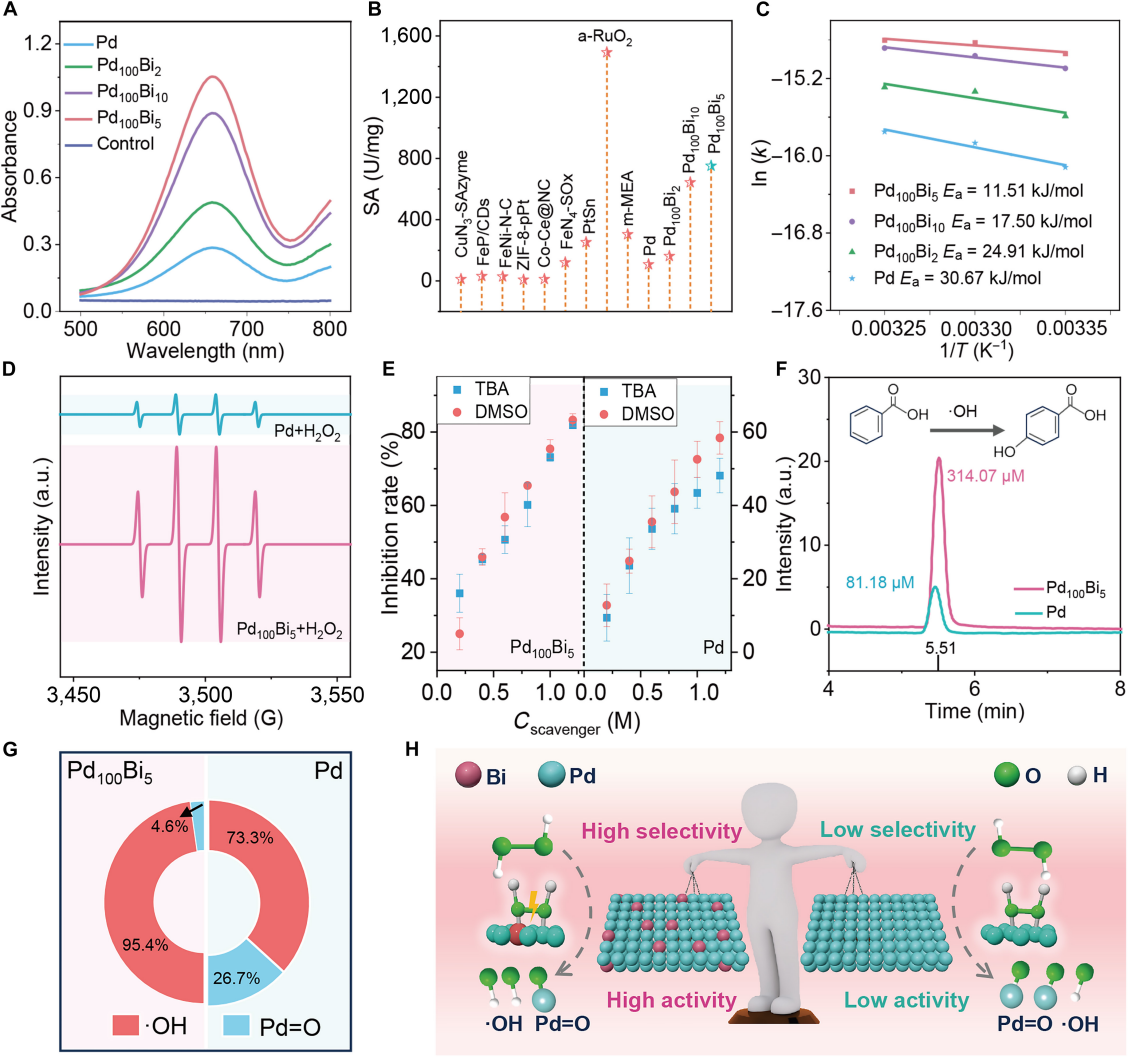
(A) Absorbance spectra of the 3,3′,5,5′-tetramethylbenzidine/H_2_O_2_ system catalyzed by Pd_100_Bi_5_ and Pd_100_Bi_x_ (x = 0, 2, 10) aerogels. (B) The specific activity (SA) of the Pd_100_Bi_x_ (x = 0, 2, 5, 10) aerogels and the reported nanomaterials. CDs, carbon dots; NC, nitrogen-doped carbon; ZIF, zeolitic imidazolate framework; m-MEA, mesoporous medium-entropy alloy. (C) Arrhenius plots of ln(*k*) versus 1/*T* for H_2_O_2_ activation ability of Pd_100_Bi_x_ (x = 0, 2, 5, 10). *E*_a_, activation energy. (D) 5,5-Dimethyl-1-pyrrolidine-*N*-oxide spin-trapping electron-paramagnetic-resonance spectra of Pd aerogels with H_2_O_2_ and Pd_100_Bi_5_ aerogels with H_2_O_2_. (E) The inhibited activity of both Pd_100_Bi_5_ and Pd systems after treatment with various concentrations of scavengers, including *tert*-butyl alcohol (TBA) and dimethylsulfoxide (DMSO). (F) Concentration of •OH concentration between the Pd and PdBi systems. The diagram illustrates the reaction mechanism of benzoic acid (BA) with •OH, resulting in the formation of *p*-hydroxybenzoic acid. (G) Selectivity of active intermediates in Pd_100_Bi_5_ and Pd-mediated H_2_O_2_ activation. (H) Schematic illustration of selective H_2_O_2_ activation on the Pd_100_Bi_5_ aerogels.

The ability of Pd_100_Bi_x_ (x = 2, 5, 10) aerogels to activate H_2_O_2_ was examined using 3,3′,5,5′-tetramethylbenzidine (TMB) as a substrate, which is quantitatively assessed by measuring the absorbance of the oxidized form of TMB (oxTMB) at 652 nm [28,29]. As expected, Pd_100_Bi_x_ (x = 2, 5, 10) aerogels have a higher absorbance value than Pd aerogels, demonstrating excellent H_2_O_2_ activation ability, indicating that the p–d orbital hybridization of Pd_100_Bi_x_ (x = 2, 5, 10) aerogels plays a critical role in enhancing the H_2_O_2_ activation ability (Fig. 3A). Among them, Pd_100_Bi_x_ (x = 2, 10) aerogels are optimized for H_2_O_2_ activation. The specific activity (SA) is a quantitative measure of the H_2_O_2_-activation ability of Pd_100_Bi_x_ (x = 2, 5, 10) aerogels. Pd_100_Bi_5_ aerogels demonstrate a high SA value (751.1 U/mg), exceeding those of Pd_100_Bi_10_ (642.8 U/mg), Pd_100_Bi_2_ (161.4 U/mg), and Pd aerogels (107.6 U/mg) (Fig. 3B and Fig. S6). It should be pointed out that the Pd_100_Bi_10_ aerogels, despite having the largest tensile strain, exhibit a lower H_2_O_2_ activation performance (SA = 642.8 U/mg) compared with Pd_100_Bi_5_ (751.1 U/mg). This indicates that the simple strain effect is not sufficient to optimize the catalytic activity. We believe that the enhanced p–d orbital hybridization due to the moderate Bi doping plays a more crucial role in regulating the electrons, enhancing the H₂O₂ adsorption, and promoting the subsequent cascade hybridization at the core of the Pd d-band center. Besides, the kinetics of Pd and PdBi aerogels are evaluated using the Michaelis–Menten equation (Fig. S7 and Table S2). Pd_100_Bi_5_ aerogels exhibit lower *K*_m_ values and higher *V*_max_ values compared to Pd_100_Bi_x_ (x = 0, 2, 10), indicating stronger substrate affinity and superior catalytic performance. Then, cyclic voltammetry is performed to assess the catalytic efficiency of H_2_O_2_ using Pd and Pd_100_Bi_5_ aerogel-modified glassy carbon electrodes in an N_2_-saturated acetate buffer. As shown in Fig. S8, Pd_100_Bi_5_ aerogels exhibit a notable reduction in current, indicating exceptional H_2_O_2_ reduction activity. To further reveal the mechanism of H_2_O_2_ activation enhanced by Pd_100_Bi_5_ aerogels, the activation energy (*E*_a_) of H_2_O_2_ activation ability is compared using the Arrhenius equation [30]. The *E*_a_ value for the H_2_O_2_ activation of Pd_100_Bi_5_ aerogels (11.51 kJ/mol) is lower than Pd_100_Bi_10_ (17.50 kJ/mol), Pd_100_Bi_2_ (24.91 kJ/mol), and Pd aerogels (30.67 kJ/mol), indicating that Pd_100_Bi_5_ aerogels can more easily activate H_2_O_2_ (Fig. 3C). This value is comparable to those reported for other nanozymes, further confirming the excellent activation ability of Pd_100_Bi_5_ aerogels (Table S3). To achieve a more comprehensive understanding of the fundamental mechanism driving the enhanced H_2_O_2_ activation capability of Pd_100_Bi_5_ aerogels, the intermediates were investigated. A standard fluorescent probe, terephthalic acid, can capture •OH, yielding a fluorescent product with an emission peak at 440 nm. Upon the concurrent presence of Pd_100_Bi_5_ aerogels, H_2_O_2_, and terephthalic acid, a conspicuous fluorescence signal emerges, affirming the presence of •OH intermediates (Fig. S9). Electron-paramagnetic-resonance measurements applying 5,5-dimethyl-1-pyrrolidine-*N*-oxide (DMPO) as the spin-trapping agent were further performed, as shown in Fig. 3D. The Pd_100_Bi_5_ aerogels–H_2_O_2_ system exhibits a more pronounced DMPO–•OH signal than Pd aerogels, indicating that Pd_100_Bi_5_ aerogels activate H_2_O_2_ to generate more •OH. Based on this, to comprehensively determine whether there are other types of ROS in the system, using DMPO and 2,2,6,6-tetramethylpiperidinooxy as traps, respectively, no characteristic signals of superoxide anion radicals (•O_2_^−^) and singlet oxygen (^1^O_2_) were detected, thereby excluding the interference of these 2 common ROS (Fig. S10). In addition, in situ Fourier-transform infrared spectroscopy is used to verify the changes in chemical bonds and functional groups during the activation of H_2_O_2_ on Pd aerogels and Pd_100_Bi_5_ aerogels [31,32]. The surface of the Pd aerogels displays distinct peaks at 1,230 and 1,650 cm^−1^, which can be attributed to the bending vibrations of •OH and H_2_O, respectively. In Pd_100_Bi_5_ aerogels, the H_2_O bending vibrational peak is importantly enhanced, indicating a higher efficiency of activated H_2_O_2_. It is noteworthy that, in comparison to Pd aerogels, a bending vibration peak of the O–O bond at 1,125 cm^−1^ is observed in PdBi aerogels. This suggests that the PdBi aerogels effectively promote the polarized elongation of the O–O bond, thereby facilitating the activation of H_2_O_2_ for •OH generation (Fig. S11) [33]. To determine the proportion of •OH, a series of scavengers, including *tert*-butyl alcohol (TBA, •OH scavengers) and dimethylsulfoxide (DMSO, both •OH and metal-oxo species scavengers), are introduced into the H_2_O_2_ activation process system [34,35].

As depicted in Fig. S12 and Fig. 3E, the inhibitory effects of TBA and DMSO on the activation of H_2_O_2_ by Pd_100_Bi_5_ are similar, indicating that Pd_100_Bi_5_ mainly activates H_2_O_2_ to generate •OH. In contrast to Pd_100_Bi_5_, in the Pd system for H_2_O_2_ activation, the inhibitory effect of DMSO is stronger than that of TBA, suggesting that in the Pd system, H_2_O_2_ generates •OH and metal-oxo. Further analysis suggests that Pd_100_Bi_5_ aerogels decreased metal-oxo selectivity from 23.3% to 1.70%, dramatically increasing the •OH selectivity from 76.7% to 98.3%. Using benzoic acid (BA) as the probe molecule, a specific hydroxylation reaction converts BA into *p*-hydroxybenzoic acid. We observe that the concentration of •OH in the Pd system is 81.18 μmol/l, while the production of •OH in the Pd_100_Bi_5_ system increased to 314.07 μmol/l. Compared with Pd (73.3%), Pd_100_Bi_5_ (95.4%) importantly enhances the selectivity of •OH during the activation of H_2_O_2_ (Fig. S13 and Fig. 3F and G). This result is basically consistent with the results of the quenching experiment with the scavenger. In summary, Pd_100_Bi_5_ aerogels can selectively activate H_2_O_2_ to produce •OH and enhance H_2_O_2_ activation ability (Fig. 3H). Additionally, the stability of Pd_100_Bi_5_ aerogels is assessed. Pd_100_Bi_5_ aerogels have excellent tolerance and heat resistance (Figs. S14 and S15). Furthermore, the cyclic stability test demonstrates that after 10 cycles, the Pd_100_Bi_5_ aerogels still maintained excellent activity (Fig. S16). To further verify the structural integrity and chemical stability of the Pd_100_Bi_5_ after the reaction, we conduct a series of characterization tests on the nanozyme after the reaction. The TEM and x-ray diffraction results show that the Pd_100_Bi_5_ aerogel after the reaction still maintains its original porous framework structure and crystal phase composition, which is consistent with the state before the reaction (Fig. S17A to C). The XPS analysis indicated that the surface electronic states of Pd and Bi did not undergo important changes, indicating that the chemical state of the catalyst was maintained during the reaction process (Fig. S17D and E). Additionally, inductively coupled plasma tests on the supernatant after the reaction did not detect any leakage of Pd ions or Bi ions, further ruling out the possibility of the active components leaching out during the reaction. The above results collectively confirm that the Pd_100_Bi_5_ aerogel has excellent structural and chemical stability under catalytic reaction conditions (Table S4).

### Intermolecular orbital hybridization between the Pd atom in PdBi and H_2_O_2_

**Fig. 4.**
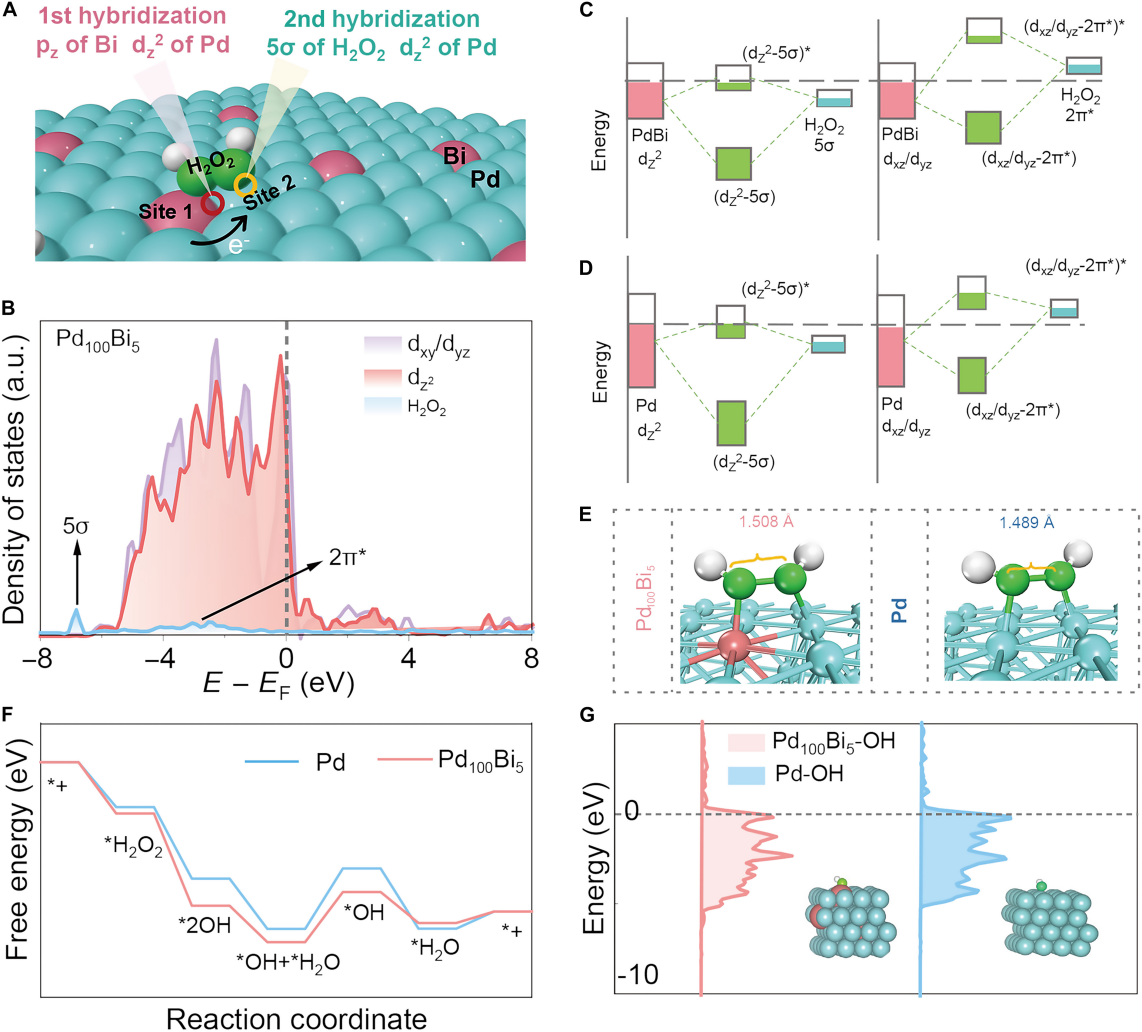
(A) Schematic illustration of orbital interactions between H_2_O_2_ (5σ and 2π*) and 4d orbital for (B) Pd_100_Bi_5_ aerogels. (B) The DOS for Pd 4d d_xy_/d_yz_/d_z_^2^ orbitals and adsorbed H_2_O_2_ for Pd_100_Bi_5_ aerogels. Schematic illustration of orbital interactions between H_2_O_2_ (5σ and 2π*) and 4d orbital for (C) Pd_100_Bi_5_ and (D) Pd aerogels. (E) Structures of adsorbed H_2_O_2_ on Pd aerogels and Pd_100_Bi_5_ aerogels. (F) The energy-change diagram of the H_2_O_2_ reduction process on Pd aerogels and Pd_100_Bi_5_ aerogels. (G) The center of the d orbital of Pd after hybridization with the H_2_O_2_ cascade orbitals. Blue, red, white, and green spheres in the insets represent Pd, Bi, H, and O atoms, respectively.

To further clarify the mechanism of H_2_O_2_ activation and explain the outstanding catalytic performance of Pd_100_Bi_5_ aerogels, we propose a cascade hybridization model (p–d–σ), involving site 2 (p-block), site 1 (d-block), and hydrogen peroxide (σ). By precisely controlling the p–d orbital hybridization between sites 1 and 2, specific orbital coupling is expected to be achieved in the subsequent cascade hybridization. Firstly, given that HR-TEM and SAED images confirm (111) as the dominant exposed facet in the Pd and PdBi aerogels, we employ the Pd(111) surface as a representative model to investigate the effect of Bi doping on the electronic structure and adsorption energetics (Fig. S18). Due to the strong orbital hybridization between the d orbitals of Pd and the p orbitals of Bi, the d orbitals of Pd after hybridization have a greater degree of overlap with the bonding and antibonding orbitals when hybridizing with H_2_O_2_ (Fig. 4B and Fig. S19). Specifically, the energy levels of the 4d_z_^2^ and 4d_xz/yz_ orbitals of Pd atoms in Pd_100_Bi_5_ are higher than those in Pd aerogels after p–d hybridization. Therefore, when the 4d orbitals of Pd in Pd_100_Bi_5_ interact with the 5σ and 2π* orbitals of H_2_O_2_, the energy of the antibonding orbitals is higher than that in Pd. This results in an increased electron occupancy of the bonding orbitals, thereby strengthening the adsorption interaction of H_2_O_2_ on Pd_100_Bi_5_ and weakening it on Pd (Fig. 4C and D) [36,37]. Further hybridization between Pd (4d_z_^2^, 4d_xy_, and 4d_yz_) and H_2_O_2_ (5σ and 2π*) in Pd_100_Bi_5_ aerogels is referred to as cascade orbital hybridization, which can achieve highly efficient and selective activation of H_2_O_2_ into •OH. Furthermore, to verify the role of the cascade orbital hybridization in the selectivity and efficiency of H_2_O_2_ activation, the lengths of H_2_O_2_ adsorbed on the surfaces of Pd_100_Bi_5_ aerogels and Pd aerogels were also calculated. To be specific, the O–O bonding length of the adsorbent of H_2_O_2_ (1.508 Å) on PdBi aerogels is longer, while the adsorbent of the O–O bonding length of H_2_O_2_ (1.489 Å) on Pd is shorter (Fig. 4E). Therefore, compared with Pd aerogels, Pd_100_Bi_5_ aerogels are more conducive to the selective generation of •OH from O–O bond breaking in H_2_O_2_. In addition, as shown in Fig. 4F and Fig. S20, the activation process and energy-change diagram of H_2_O_2_ on the Pd_100_Bi_5_ aerogels and Pd aerogels are presented. Firstly, the free energy of *H_2_O_2_ on Pd_100_Bi_5_ aerogels is significantly lower than that on Pd aerogels, which can promote H_2_O_2_ adsorption and match well with the PDOS analysis, providing evidence for promoting H_2_O_2_ activation through cascade orbital hybridization. Besides, *H_2_O_2_ is cleaved into 2 adsorbed *OH on Pd and Bi sites. Next, *OH is protonated to form *H_2_O. Finally, *H_2_O is desorbed in succession, and the Pd_100_Bi_5_ aerogels return to the initial state. In this reaction, the step from *OH and *H_2_O to *OH can be regarded as the rate-determining step. The energy barrier for the rate-determining step of Pd_100_Bi_5_ aerogels (1.279 eV) is lower than that of Pd aerogels (1.543 eV), which is attributed to the redistribution of the electronic structure after the hybridization of Pd_100_Bi_5_ aerogels and H_2_O_2_ cascade orbitals. After the occurrence of the cascade orbital hybridization, the d-band center of Pd in the Pd_100_Bi_5_ aerogels (−2.42 eV) is lowered relative to the d-band center of pure Pd aerogels (−1.23 eV), which is conducive to the desorption of water molecules on the Pd_100_Bi_5_ aerogels (Fig. 4G). In summary, the p–d orbital hybridization interaction between Pd and Bi plays a crucial role in amplifying the cascade orbital hybridization process between Pd and H_2_O_2_. This interaction promotes the cascade orbital hybridization between Pd and H_2_O_2_, leading to the redistribution of electrons on the surface of the Pd aerogels, thereby facilitating the cleavage of the O–O bond during H_2_O_2_ activation and the effective removal of H_2_O. As a result, this cascade orbital hybridization importantly enhances the selectivity and efficiency of H_2_O_2_ activation.

### Identification of different pesticide residues

In agriculture, various pesticides, including organophosphorus, organochlorine, carbamates, phenol carboxylic acids, and sulfonamides, are widely used as growth regulators. However, the incomplete degradation of these pesticides leads to residues that pose important threats to food safety. Therefore, simultaneously detecting and differentiating pesticides with diverse structures and properties remains a key scientific issue that urgently needs to be addressed [38–43]. To address this issue, a novel sensing strategy based on the catalytic inhibition effect has been developed. This strategy enables differential recognition of various pesticide types (e.g., organophosphorus, organochlorine, and carbamate) by precisely modulating the interactions between pesticide molecules and the nanozyme’s active sites. Specifically, it shows a gradient inhibition effect on the activation process of H_2_O_2_ and the generation efficiency of •OH, thereby establishing a quantitative structure–activity relationship between the degree of inhibition and the type and concentration of pesticides.

**Fig. 5.**
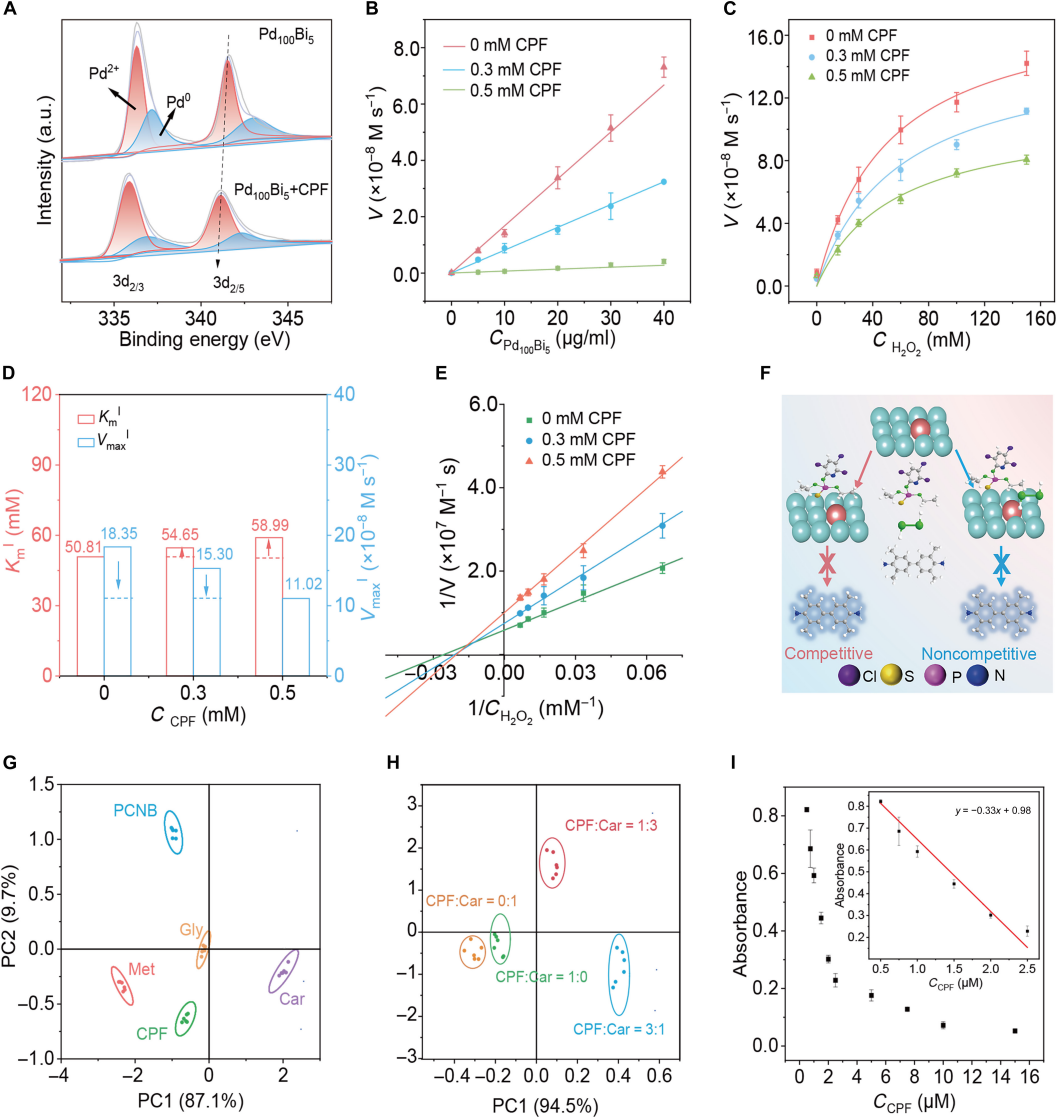
(A) XPS spectra of Pd 4d for Pd_100_Bi_5_ aerogels and Pd_100_Bi_5_ aerogels + chlorpyrifos (CPF). (B) The initial velocities at different CPF concentrations vary with the Pd_100_Bi_5_ aerogel concentration. (C) Michaelis–Menten curves of Pd_100_Bi_5_ aerogels toward various concentrations of H_2_O_2_ at different CPF concentrations. (D) The *V*_max_^I^ and *K*_m_^I^ values of Pd_100_Bi_5_ aerogels in the presence of different concentrations of CPF. (E) Lineweaver–Burk plots of Pd_100_Bi_5_ aerogels toward various concentrations of H_2_O_2_ to varying concentrations of CPF. (F) Schematic diagram of the inhibition types of CPF on Pd_100_Bi_5_ aerogels. (G) Two-dimensional (2D) canonical score plots derived from the colorimetric sensor array responses to pentachloronitrobenzene (PCNB), metsulfuron-methyl (Met), glyphosate (Gly), carbaryl (Car), Chipton, and CPF. (H) 2D canonical score plots of the sensor array in response to binary pesticide mixtures with different molar ratios. (I) Linear ranges of the detection result from CPF.

For instance, organophosphorus pesticides like chlorpyrifos (CPF) strongly adsorb onto the nanozyme surface, importantly suppressing •OH production, while other pesticides (e.g., sulfonamides or phenolic acids) exhibit weaker inhibitory effects. By capturing these varying inhibition signals, the sensor array translates them into distinct response patterns, thereby enabling the detection of different pesticide residues. CPF is employed as a representative model for pesticide molecules. As illustrated in Fig. 5A, the XPS analysis reveals that the peak of Pd 3d shifts importantly with the addition of CPF, indicating an interaction between Pd_100_Bi_5_ aerogels and CPF [44,45]. This finding highlights the need for further exploration into the inhibitory effects of CPF on Pd_100_Bi_5_ aerogels [27,46,47]. As shown in Fig. 5B, it can be seen that the rate of H_2_O_2_ activation exhibits a linear relationship with the concentration of Pd_100_Bi_5_ aerogels. As CPF increases, the slope of the line through the origin decreases, indicating that CPF reversibly inhibits the ability of Pd_100_Bi_5_ aerogels to activate H_2_O_2_. We additionally determine the initial velocity at various H_2_O_2_ concentrations in the presence of CPF, and the catalytic kinetics align with the Michaelis–Menten curve equation (Fig. 5C and Table S5). In the presence of the inhibitor CPF, the Michaelis–Menten constant is denoted as *K*_m_^I^ (inhibited state), while the maximum initial velocity is represented as *V*_max_^I^. With the increase of CPF concentration, *K*_m_^I^ increased and *V*_max_^I^ decreased (Fig. 5D). Moreover, Lineweaver–Burk plots constructed at various concentrations of CPF crossed in the second quadrant. This suggests that the inhibition is of a mixed type, encompassing both competitive and noncompetitive inhibition (Fig. 5E). *K*_I_ and *K*_I_′ represent the inhibition constants of the nanozyme–inhibitor and nanozyme–substrate–inhibitor systems, respectively. *K*_I_ and *K*_I_′ are calculated to be 0.557 and 0.783 mM. *K*_I_ < *K*_I_′ indicates that the affinity of CPF for Pd_100_Bi_5_ is higher than that of the complex formed by Pd_100_Bi_5_ and H_2_O_2_, further suggesting that competitive inhibition plays a dominant role in the mixed inhibition. In conclusion, CPF reduces the affinity of Pd_100_Bi_5_ aerogels for the substrate H_2_O_2_ through a mixed inhibition mode, thereby reducing the activation ability of H_2_O_2_ (Fig. 5F). To gain deeper insight into the molecular-scale interaction between CPF and the PdBi surface, density functional theory calculations were performed. The CPF molecule is modeled on the Pd_100_Bi_5_ surface, with the P=S functional group identified as the primary binding motif. The calculated adsorption energy of CPF on Pd_100_Bi_5_ is −0.45 eV, importantly stronger than that of H_2_O_2_ (−1.55 eV), indicating that CPF preferentially occupies the Pd active sites and competitively inhibits H_2_O_2_ activation. PDOS further shows pronounced orbital overlap between the Pd 4d orbitals and the S 3p orbitals of CPF near the Fermi level, providing direct evidence for orbital hybridization underlying the competitive inhibition mechanism (Fig. S21). In addition, other typical pesticides, including pentachloronitrobenzene (PCNB), carbaryl (Car), glyphosate (Gly), and metsulfuron-methyl (Met), are also discussed for their inhibition effects on the selective generation of •OH by activated H_2_O_2_. As shown in Fig. S22, PCNB, Car, Gly, and Met exhibit typical reversible inhibition features. According to the intersection position of the Lineweaver–Burk diagram (Fig. S23), the inhibition types of the above 5 pesticides can be determined (Table S6). Inspired by the results mentioned above, the extent of inhibition of H_2_O_2_ activation varied among different pesticide molecules, offering a promising approach to distinguish and identify multiple pesticides across diverse categories. Pd, Pd_100_Bi_2_, and Pd_100_Bi_5_ aerogels are utilized to construct a simple 3-channel sensor array. We employ sensor arrays for cross-reactive discrimination of various pesticides (CPF, PCNB, Car, Gly, and Met). Subsequently, through the application of principal component analysis for transforming the complex training-data matrix, 5 types of pesticides are effectively differentiated, resulting in tightly formed clusters (Fig. 5G). Additionally, the sensing arrays demonstrate the ability to precisely recognize mixtures of CPF and Car in various proportions (Fig. 5H). To provide a more robust and quantitative assessment of sensitivity, we perform conventional univariate calibration using the Pd_100_Bi_5_ aerogels that exhibit the highest response to CPF. As shown in Fig. 5I, the response intensity shows a linear relationship with CPF concentration in the range of 0.5 to 2.5 μM. The detection limit (0.23 μM) is calculated as 3*σ*/slope, where *σ* is the standard deviation of the blank measurements (*n* = 10). This detection limit is considerably lower than the maximum residue limits for CPF established by the European Union.

In the actual sample analysis experiment, simulated pesticide spraying is used to analyze pesticide residues on grape surfaces. Extracting solutions containing different types of pesticides as samples to be tested produces unique response signals. As shown in Fig. S24, the 2-dimensional canonical score plot shows that the 5 types of pesticides are separated into 5 distinct regions, clearly apart from each other, demonstrating excellent discriminating ability.

## Conclusion

In summary, the integration of p-block metal Bi into Pd aerogels to form PdBi aerogels has been shown to importantly enhance the selective activation of H_2_O_2_ through the introduction of tensile strain. This strain leads to a subsequent enhancement of orbital hybridization between the d orbitals of Pd and the p orbitals of Bi. This change in orbital hybridization and energy levels facilitates the interaction between the Pd 4d_xz/yz_ and 4d_z_^2^ orbitals and the 2π* and 5σ orbitals of H_2_O_2_ molecules during H_2_O_2_ activation. Consequently, Pd_100_Bi_5_ aerogels exhibit reduced metal-oxo selectivity and markedly increased •OH selectivity, thereby improving the activation efficiency of H_2_O_2_. Leveraging these properties, a colorimetric sensor array has been developed to effectively distinguish and detect various types of pesticides. Furthermore, we expect that the cascade orbital hybridization strategy demonstrated here is expected to be extendable to other catalytic processes involving small-molecule activation, such as the oxygen reduction reaction and the oxygen evolution reaction, where tuning intermediate adsorption is critical.

## Materials and Methods

### Materials

Pd(acac)_2_, BiCl_3_, PCNB, Gly, CPF, Car, Met, Chipton, and horseradish peroxidase were purchased from Shanghai Aladdin Biochemical Technology Co., Ltd. Sodium acetate (NaAc), terephthalic acid, and TMB were obtained from Sinopharm Chemical Reagent Co., Ltd.

### Preparation of Pd and PdBi aerogels

In a typical synthesis of PdBi aerogels, 2 ml of aqueous NaBH_4_ (0.05 M) was quickly injected into 30 ml of an aqueous metal precursor solution containing 0.4 ml Pd(acac)_2_ (0.1 M) and 0.04 ml BiCl_3_ (0.05 M), with stirring at 60 °C. The prepared mixture solution was stirred at 600 rpm for 1 min and changed from faint yellow to black. Finally, the black solution was heated to 60 °C for 2 h to form PdBi aerogels. The black monolith solids were carefully washed with water 3 times and finally dried at −60 °C in the lyophilized chamber. Pd and PdBi aerogels were prepared with Pd and Bi source mole ratios of 100:0, 100:2, and 100:10 using the same process.

### Evaluations of the activation properties of H_2_O_2_

The ability of Pd aerogels and PdBi aerogels to activate H_2_O_2_ was evaluated through a traditional colorimetric reaction, using TMB (a colorimetric agent) as an indicator in the presence of acetic acid–sodium acetate (HAc–NaAc) buffer and an aqueous H_2_O_2_ solution. Specifically, 5 μl of Pd aerogels or PdBi aerogels (0.05 mg/ml), 100 μl of H_2_O_2_ (100 mM) water solution, and 100 μl of TMB (1 mM) ethanol solution were sequentially added to 100 μl of HAc–NaAc buffer solution (pH 3.0, 0.1 M). Following a 2-min incubation period, the absorbance at 652 nm of the resulting mixture was measured using a multimode reader.

### Construction of the colorimetric sensor array for pesticide identification

A colorimetric sensor array based on PdBi aerogels (3 × 6 × 6) was assembled in a 96-well plate. The detailed steps were as follows: HAc–NaAc, TMB, H_2_O_2_, 3 distinct types of PdBi aerogels, and pesticide standards at various concentrations were added into the 96-well plate. Following a 5-min incubation period, the absorbance of the solution was recorded at 652 nm using a microplate reader. This procedure was conducted 6 times for 6 different antioxidants. As a result, the antioxidants were evaluated using 3 types of PdBi aerogels, generating a 3 × 6 × 6 data matrix.

## Data Availability

The data supporting this study’s findings are available within the article and its supplementary materials.
