# Supplementary material for: Bi-Doped Pd Aerogels with Tensile-Strain-Induced Cascade Orbital Hybridization Boost H2O2 Selective Activation for Efficient Pesticide Distinction
Source: Research (Wash D C). 2026 Jun 5;9:1300. doi: 10.34133/research.1300 (PMC13237496; doi:10.34133/research.1300)
Supplement: Supplementary 1 — Figs. S1 to S24 Tables S1 to S6 [file research.1300.f1.docx]

PdBi aerogels with tensile strain-induced cascade orbital hybridization boost H_2_O_2_ selective activation for efficient pesticide distinction

Ruimin Li^1,2#^, Chengjie Chen^1#^, Lijun Hu^1^, Dongbo Yan^1^, Xiaotong Li^1^, Xiangkun Jia^1^, Yu Wu^2^, Lei Jiao^1,^*, Yanling Zhai^1,^*, Chengzhou Zhu^2^^,^*

^1^ Institute of Molecular Metrology, College of Chemistry and Chemical Engineering, Qingdao University, Qingdao 266071 (P. R. China)

^2^ State Key Laboratory of Green Pesticide, International Joint Research Center for Intelligent Biosensing Technology and Health, College of Chemistry, Central China Normal University, Wuhan 430079 (P. R. China)

*Corresponding author.

E-mail: [jiaolei@qdu.edu.cn](mailto:jiaolei@qdu.edu.cn); [zhaiyanling@qdu.edu.cn](mailto:zhaiyanling@qdu.edu.cn); czzhu@ccnu.edu.cn

Table of Contents

[1. Experimental Procedures 4](#_Toc217402867)

[1.1 Apparatus 4](#_Toc217402868)

[1.2 Evaluation of reaction kinetics. 4](#_Toc217402869)

[1.3 Evaluation of Specific Activities (SA). 5](#_Toc217402870)

[1.4 Experimental details of quantification •OH. 5](#_Toc217402871)

[1.5 Density Functional Theory Calculations. 6](#_Toc217402872)

[1.6 Real sample analysis 7](#_Toc217402873)

[2. Supporting Figures. 8](#_Toc217402874)

[3. Supplementary tables 31](#_Toc217402875)

[4. Supplementary reference 34](#_Toc217402876)

1. Experimental Procedures

1.1 Apparatus

Scanning electron microscopy (SEM) images were captured by JSM-78OOF. Transmission electron microscopy (TEM), selected-area electron diffraction (SAED), and high-resolution transmission electron microscopy (HRTEM) images were captured by JEOL JEM F200. X-ray photoelectron spectroscopy (XPS) measurements were performed on a Thermo Scientific K-Alpha. Crystal structures of samples were examined by an X-ray diffractometer (XRD) (Rigaku Smartlab SE). Electron paramagnetic resonance (EPR) spectra were from Bruker A300. All enzyme kinetics data and UV-vis spectra were performed by a multimode reader (Tecan Spark, Switzerland).

1.2 Lattice strain calculation

Firstly, using pure Pd as the standard sample, the instrument zero offset was calculated. By comparing the measured peak positions of pure Pd with the standard values on the PDF card (PDF #46-1043), the average offset of 0.285 was obtained, which was used to correct the measured diffraction peak positions of all PdBi samples. Secondly, for each PdBi sample, the (111) diffraction peak was selected, and the corrected interplanar spacing dhkl was calculated:

$$\text{d}_{\text{hkl}}\text{=}\frac{\text{λ}}{\text{2 sin}\text{θ}_{\text{hkl}}}$$

Using Cu Kα radiation (λ = 0.15418 nm), hkl represents the crystal plane index, and θ_hkl_ is the corrected Bragg angle.

Subsequently, the lattice constant a_hkl_ was calculated:

$$a_{hkl}=d_{hkl}\sqrt{h^{2}+k^{2}+l^{2}}$$

Finally, the lattice strain (ε) was obtained:

$$\varepsilon=\frac{a_{sample}-a_{Pd}}{a_{Pd}}$$

1.3 Evaluation of reaction kinetics

The kinetics of Pd aerogels and PdBi aerogels were evaluated separately. First, for the kinetic data of H_2_O_2_, Pd aerogels or PdBi aerogels (5 µL, 0.05 mg/mL), an aqueous solution of H_2_O_2_ at different concentrations, TMB (100 µL, 1 mM), and alcohol solution were added into the HAc-NaAc buffer solution (100 µL, pH = 3.0, 0.1 M). Second, for the kinetic data of TMB, Pd aerogels or PdBi aerogels (5 µL, 0.05 mg/mL), H_2_O_2_ (100 µL, 100 mM) aqueous solution, different concentrations of TMB in ethanol solution were added into the HAc-NaAc buffer solution (100 µL, pH = 3.0, 0.1 M). Finally, the obtained kinetic data were evaluated using the Michaelis-Menten equation:

*V*=*V*_max_[S]/(*K*_m_+[S])

Where *V*_max_ is the maximum reaction velocity, [S] is the concentration of the substrate, *K*_m_ is the Michaelis−Menten constant, and V is the initial velocity.

1.4 Evaluation of Specific Activities (SA).

First, different concentrations of Pd aerogels or PdBi aerogels, H_2_O_2_ (100 µL, 10 M) aqueous solution, and TMB (100 µL, 10 mg/mL) DMSO solution were added into the HAc-NaAc buffer solution (100 µL, pH = 3.0, 0.1 M). Second, the above-mixed solutions were tested for kinetic data. Finally, the SA was calculated by the following equation:

SA= [V/(Ԑ×l) ×(∆A/∆t)]/m

Where V is the total volume of the solution (μL); Ԑ is the molar absorption coefficient of TMB (39,000 M^-1^ cm^-1^); l is the path length of light propagation (cm); ΔA/Δt is the initial rate of change in absorbance; m is the weight (mg) of Pd aerogels and PdBi aerogels.

1.5 Experimental details of quantification •OH.

Benzoic acid (BA) was used as a probe molecule to react with •OH in catalysts-H_2_O_2_ suspension under an anaerobic environment. Briefly, 2 mg of catalyst was added to a round-bottomed flask with a rubber stopper and followed by N_2_ gas bubbling to remove oxygen. Subsequently, 200 mL of BA solution (10.4 mmol/L) was continuously bubbled with N_2_ gas for 30 min to ensure an oxygen-free environment and then pipetted into the round-bottomed flask with a rubber stopper by an injection syringe. Then a certain concentration of H_2_O_2_ (Final concentration: 300 μM) was pipetted to trigger the reaction under shaking throughout the experiment. The reaction solution was collected at a predetermined time and filtered through 0.22 μm membranes. High Performance Liquid Chromatography (HPLC) was used to determine the p-hydroxybenzoic acid (p-HBA) concentration. The mobile phase was acetonitrile/water (30/70, v:v) and the detection wavelength was 270 nm. Injection volume, flow rate of mobile phase, and column temperature were fixed at 10 μL, 1.0 mL/min, and 35 °C, respectively. Given that 5.87±0.18 moles •OH reacting with BA produced one mole p-HBA. The cumulative •OH concentration is therefore approximately 5.87 times that of p-HBA concentration.

1.6 Density Functional Theory Calculations.

We have employed the Vienna Ab Initio Package (VASP) to perform all the density functional theory (DFT) calculations within the generalized gradient approximation (GGA) using the PBE formulation.[1, 2] [3] We have chosen the projected augmented wave (PAW) potentials5, 6 to describe the ionic cores and take valence electrons into account using a plane wave basis set with a kinetic energy cutoff of 520 eV. Partial occupancies of the Kohn−Sham orbitals were allowed using the Gaussian smearing method and a width of 0.05 eV.[4, 5] The electronic energy was considered self-consistent when the energy change was smaller than 10^−5^ eV. A geometry optimization was considered convergent when the force change was smaller than 0.05 eV/Å. Grimme’s DFT-D3 methodology was used to describe the dispersion interactions.[6] During structural optimizations, the Γ point in the Brillouin zone was used for k-point sampling, and all atoms were allowed to relax. The free energy of a gas phase molecule or an adsorbate on the surface was calculated by the equation G=E+ZPE−TS, where E is the total energy, ZPE is the zero-point energy, T is the temperature in kelvin (298.15 K is set here), and S is the entropy. The reported standard hydrogen electrode (SHE) model (Nørskov J K, Rossmeisl J, Logadottir A, et al. Origin of the overpotential for oxygen reduction at a fuel-cell cathode. J Phys Chem B 2004; 108: 17886-17892.) was adopted in the calculations of Gibbs free energy changes (ΔG) of all reaction steps, which were used to evaluate the reaction barrier. The chemical potential of a proton-electron pair, µ(H^+^)+µ(e^−^), is equal to half of the chemical potential of one gaseous hydrogen molecule, 1/2µ(H_2_), at U=0 V vs SHE at pH=0.

1.7 Establishment of the PdBi model

The Pd(111) surface was constructed as a three-layer slab model from the bulk fcc Pd structure (space group Fm-3m). A vacuum spacing of 15 Å was introduced along the z-direction to avoid interactions between adjacent slabs. The Bi-Pd(111) surfaces were generated by randomly replacing Pd atoms with Bi atoms, yielding Pd: Bi atomic ratios of 20:1, respectively. In all calculations, the bottom layer was constrained to the bulk geometry, while the upper two layers were allowed to relax.

1.8 Real sample analysis

Grapes were purchased from the local supermarket and were used to validate the performance of the sensor arrays. Initially, we sprayed different pesticide standard solutions (0.5 mM) evenly on the sample surface and dried them in a ventilated place for 24 hours to simulate the actual situation. Then, flush the samples with water and collect the solution to be tested.

2. Supporting Figs.


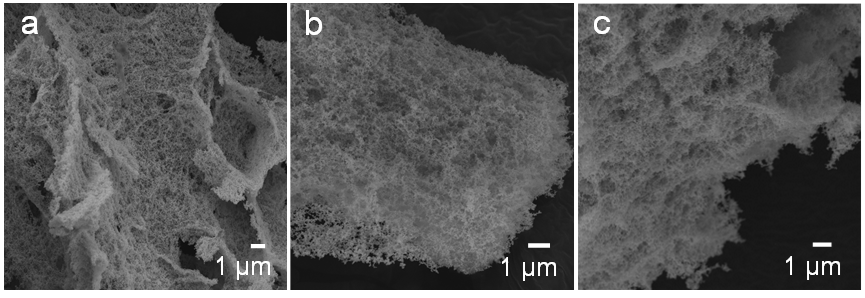


**Figs. 1**. SEM images of (a) Pd aerogels, (b) Pd_100_Bi_2,_ and (c) Pd_100_Bi_10_ aerogels.


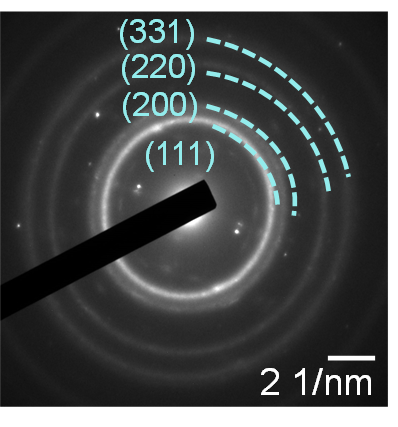


**Fig. 2**. SAED images of Pd_100_Bi_5_ aerogels.


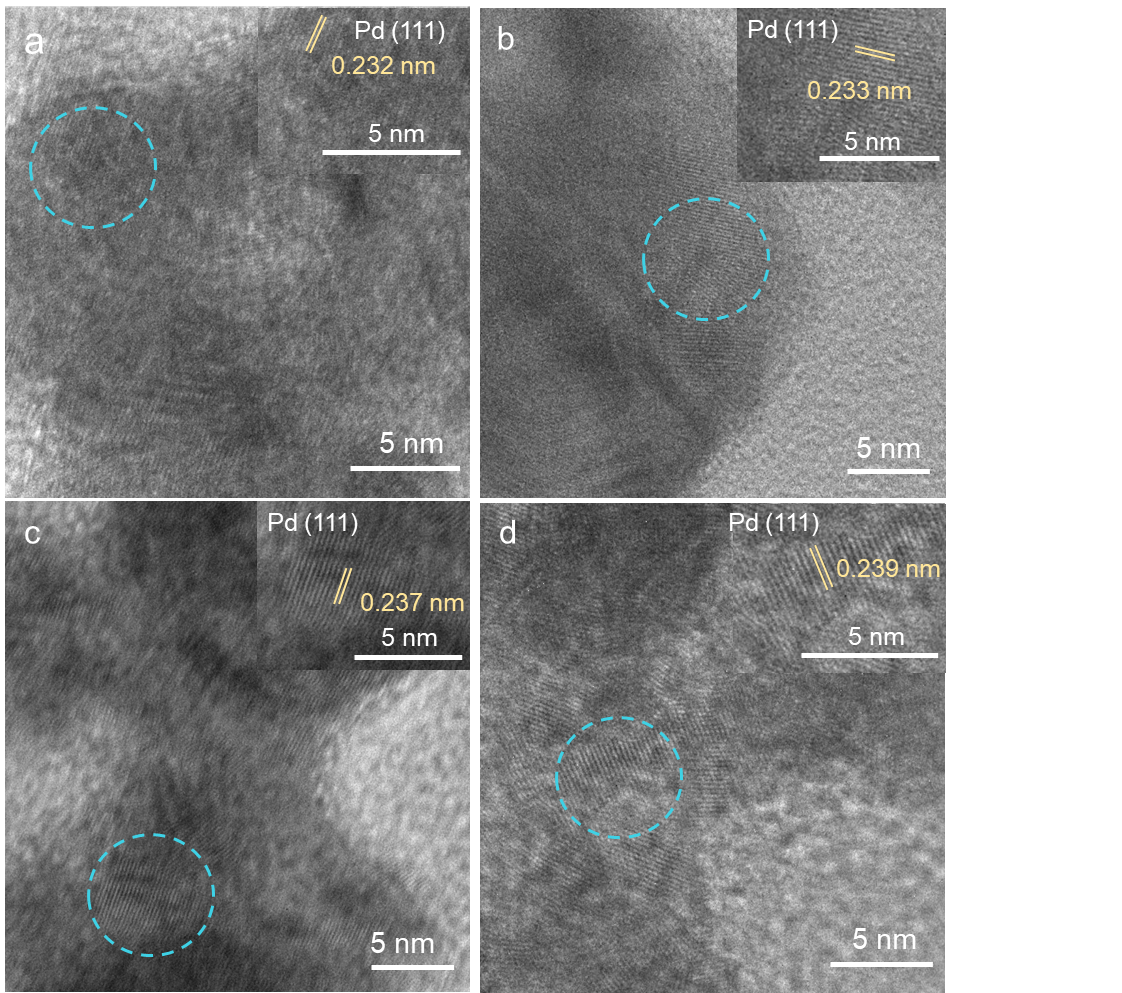


**Figs. 3**. TEM images of (a) Pd aerogels, (b) Pd_100_Bi_2_ aerogels, (c) Pd_100_Bi_5_ aerogels, and (d) Pd_100_Bi_10_ aerogels.

**Fig. 4**. DOSs of Pd d_z_^2^ and d_xz_/d_yz_ orbitals and Bi p_x_, p_y_, and p_z_ orbitals after d-p orbital hybridization.

**Fig. 5**. The calculated d-band center of surface Pd for Pd, Pd_100_Bi_2_, Pd_100_Bi_5_, and Pd_100_Bi_10_.

**Fig. 6**. Specific activity of Pd and PdBi aerogels.

**Fig. 7**. Kinetic curves of Pd and PdBi aerogels toward H_2_O_2_.

**Fig. 8**. Cyclic Voltammetry curves of the Pd and Pd_100_Bi_5_ aerogels with H_2_O_2_ and without H_2_O_2_.

**Fig. 9**. Photoluminescence spectral changes of TPA solution under simulated sunlight irradiation (excitation at 315 nm).

**Fig. 10**. EPR spectra of TEMP+^1^O_2_ and DMPO+•O_2_^-^ for the reaction of Pd_100_Bi_5_ with H_2_O_2_.

**Fig. 11**. *In situ* FT-IR spectra for (a) Pd_100_Bi_5_ and (b) Pd aerogels during the reaction of H_2_O_2_.

**Figs. 12**. (a) Absorbance of both Pd and Pd_100_Bi_5_ aerogels after treatment with various concentrations of scavengers, including TBA and DMSO. (b) Selectivity of active intermediates in Pd_100_Bi_5_ and Pd-mediated H_2_O_2_ activation.

**Figs. 13**. (a) Absorption spectra of H_2_O_2_ at different concentrations. (b) Calibration curve of H_2_O_2_ concentration-absorbance. (c) Absorption spectra of the remaining hydrogen peroxide after the reaction. (d) Concentration distribution diagrams of activated and unactivated H_2_O_2_.

(1) The standard curve of H_2_O_2_: HRP (0.01 mg/ml, 3 µL), NaAc-HAc (pH=3, 0.1M, 100 µL), different concentrations of H_2_O_2_ standard solutions (100 µL), and TMB (1 mM, 100 µL) were added to the wells of the plate in sequence. Record the absorbance values within the range of 500-800 nm. Calibrate and fit the absorbance values at 652 nm to plot the standard curve.

(2) Determination of H_2_O_2_ concentration: The concentration of the reaction was controlled to be consistent with the quantification of •OH through HLCP. Then, the post-reaction solution was taken, and HRP (0.01 mg/ml, 3 µL) and TMB (1 mM, 100 µL) were added to the well plate in sequence. The absorbance value was measured and recorded, and the remaining H_2_O_2_ concentration was obtained through the H_2_O_2_ standard curve.

(3) Calculation details of •OH selectivity: First, based on the initial concentration of H_2_O_2_ added and its standard curve, the remaining concentration of H_2_O_2_ after the reaction is calculated, thereby obtaining the total amount of H_2_O_2_ involved in the reaction.

Second, according to the concentration of p-HBA measured by HLCP, combined with the stoichiometric relationship of the reaction between hydroxyl and BA (5.87 ± 0.18 moles of hydroxyl generate 1 mole of p-HBA), the generation concentration of •OH is calculated. Then, based on the generation ratio of •OH to H_2_O_2_ (2:1), the consumption of H_2_O_2_ used for generating •OH is estimated.

Finally, by subtracting the consumption of H_2_O_2_ used for generating •OH from the total amount of H_2_O_2_ involved in the reaction, the amount of H_2_O_2_ used for generating M=O is obtained. According to the stoichiometric relationship between M=O and H_2_O_2_ (1:1), the amount of M=O is calculated.

The selectivity of oxidative activation H_2_O_2_ (to M=O) and reductive activation H_2_O_2_ (to •OH) was calculated by the following eq. S1 and eq. S2, respectively.

Selectivity to (•OH) (%) = $\frac{\left[ \text{•OH} \right]}{\left[ \text{•OH} \right]\text{+}\left[ \text{M=O} \right]}\text{*100}$ eq. S1

Selectivity to (M=O) (%) = $\frac{\left[ \text{M=O} \right]}{\left[ \text{•OH} \right]\text{+}\left[ \text{M=O} \right]}\text{*100}$ eq. S2

[M=O] and [•OH] referred to the concentrations of generated M=O and •OH, respectively.

**Fig. 14**. The effects of temperature on the activation of H_2_O_2_ by Pd_100_Bi_5_ aerogels and HRP, respectively.

**Fig. 15**. The durability of the PdBi aerogels and HRP treated with 0.01 M KOH and 0.01 M HCl.

**Fig. 16**. Stability of H_2_O_2_ activation of Pd_100_Bi_5_.

**Figs. 17**. (a) TMB (b) HRTEM and (c) XRD of PdBi aerogels after the reaction. (d) Pd 3d and (e) Bi 4f XPS spectra of Pd_100_Bi_5_ before and after the reaction.

**Figs. 18**. Pd (111), Pd_100_Bi_2_ (111), Pd_100_Bi_5_ (111), and Pd_100_Bi_10_ (111) models. Blue and red spheres in the insets represent Pd and Bi atoms, respectively.

**Fig. 19**. The DOS for Pd 4d d_xy_/d_yz_ /d_z_^2^ orbitals and adsorbed H_2_O_2_ orbitals for Pd aerogels.

**
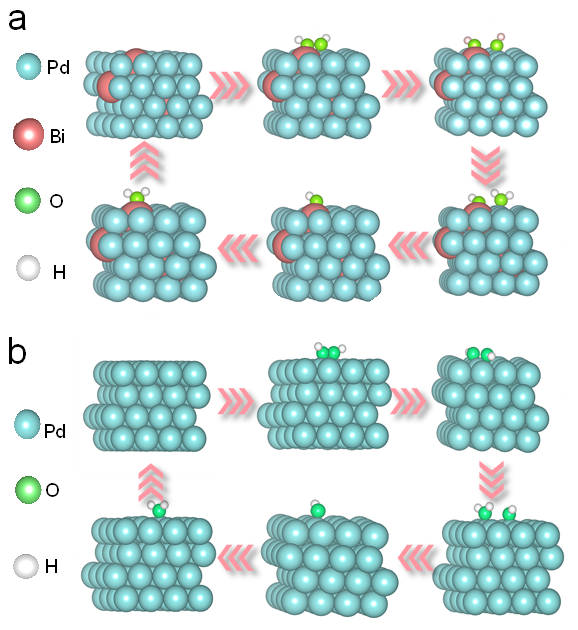
**

**Figs. 20**. Schematic diagram of the elementary step of reaction on (a) Pd_100_Bi_5_ aerogels and (b) Pd aerogels.

**Figs. 21**. Pd_100_Bi_5_ aerogels adsorb (a) the CPF model. (b) The DOS for Pd 4d orbitals and adsorbed CPF for Pd_100_Bi_5_ aerogels.

**Figs. 22**. The initial velocities at different (a) PCNB, (b) Car, (c) Gly, and (d) Met concentrations vary with Pd_100_Bi_5_ aerogels concentration.

**Figs. 23**. Lineweaver-Burk plots of Pd_100_Bi_5_ aerogels towards various concentrations of H_2_O_2_ at different concentrations of (a) PCNB, (b) Car, (c) Gly, and (d) Met.

**Figs. 24**. Discrimination and identification of pesticides in real samples. PCA score plots for discriminating five pesticides (CPF, Gly, Car, Met, and PCNB) in Grapes.

3. Supplementary tables

**Table 1**. The composition of the Pd aerogels, Pd_100_Bi_2_ aerogels, Pd_100_Bi_5_ aerogels, and Pd_100_Bi_10_ aerogels by ICP-OES.

| Samples | Pd (atom%) | Bi (atom%) |
| --- | --- | --- |
| Pd | 100 | 0 |
| Pd_100_Bi_2_ | 97.9 | 2.1 |
| Pd_100_Bi_5_ | 95.8 | 4.2 |
| Pd_100_Bi_10_ | 90.4 | 9.6 |

**Table 2**. *V*_max_ and *K*_m_ of PdBi aerogels and Pd aerogels.

|  | Substrate | *V*_max_ (M s^-1^) | *K*_m_ |
| --- | --- | --- | --- |
| Pd | H_2_O_2_ | 0.815×10^-7^ | 43.26 |
| Pd_100_Bi_2_ | H_2_O_2_ | 2.849×10^-7^ | 43.315 |
| Pd_100_Bi_5_ | H_2_O_2_ | 5.044×10^-7^ | 35.233 |
| Pd_100_Bi_10_ | H_2_O_2_ | 4.7937×10^-7^ | 35.492 |

**Table 3**. Comparison of activation energies of representative nanozyme systems.

| Nanozyme | Ea (KJ/mol) | Reference |
| --- | --- | --- |
| MOF-His-Cu | 19.46 | [7] |
| Cu_SA_-AuCu_NPs_/NC | 1.39 | [8] |
| PtPdRhNi | 19.04 | [9] |
| ZIF-8-pPt | 7.09 | [10] |
| Fe-N_3_S | 11.94 | [11] |
| Fe-S/N-C | 10.31 | [12] |
| Pd | 30.67 | This work |
| Pd_100_Bi_2_ | 24.91 |  |
| Pd_100_Bi_10_ | 17.50 |  |
| Pd_100_Bi_5_ | 11.51 |  |

**Table 4**. The ICP results of the supernatant solutions before and after the reaction of Pd_100_Bi_5_ aerogels.

| Sample | Pd (w%) |
| --- | --- |
| Before | 0.017 |
| After | 0.019 |

**Table 5**. Kinetic parameters of Pd_100_Bi_5_ aerogels toward H_2_O_2_ at different CPF concentrations.

| CPF concentration (mM) | *V*_max_^I^ (M s^-1^) | *K*_m_^I^ |
| --- | --- | --- |
| 0 | 1.8346×10^-7^ | 50.81 |
| 0.3 | 1.5304×10^-7^ | 54.65 |
| 0.5 | 1.1023×10^-7^ | 58.99 |

**Table 6**. Inhibition types of different pesticides.

|  | Inhibitory type | Specific inhibition type |
| --- | --- | --- |
| Car | Reversible | Noncompetitive and competitive |
| Met | Reversible | Competitive |
| PCNB | Reversible | Anticompetitive and noncompetitive |
| Gly | Reversible | Noncompetitive and competitive |

4. Supplementary reference

[1] G. Kresse, J. Furthmüller, Efficiency of ab-initio total energy calculations for metals and semiconductors using a plane-wave basis set, Comput. Mater. Sci., 6 (1996) 15-50.

[2] G. Kresse, J. Furthmüller, Efficient iterative schemes for ab initio total-energy calculations using a plane-wave basis set, Phys. Rev. B, 54 (1996) 11169-11186.

[3] J.P. Perdew, K. Burke, M. Ernzerhof, Generalized Gradient Approximation Made Simple, Phys. Rev. Lett., 77 (1996) 3865-3868.

[4] G. Kresse, D. Joubert, From ultrasoft pseudopotentials to the projector augmented-wave method, Phys. Rev. B, 59 (1999) 1758-1775.

[5] P.E. Blöchl, Projector augmented-wave method, Phys. Rev. B, 50 (1994) 17953-17979.

[6] S. Grimme, J. Antony, S. Ehrlich, H. Krieg, A consistent and accurate ab initio parametrization of density functional dispersion correction (DFT-D) for the 94 elements H-Pu, J. Chem. Phys., 132 (2010) 154104.

[7] M. Sha, L. Rao, W. Xu, Y. Qin, R. Su, Y. Wu, Q. Fang, H. Wang, X. Cui, L. Zheng, W. Gu, C. Zhu, Amino-Ligand-Coordinated Dicopper Active Sites Enable Catechol Oxidase-Like Activity for Chiral Recognition and Catalysis, Nano Lett., 23 (2023) 701-709.

[8] X. Jia, L. Jiao, R. Li, C. Chen, X. Li, L. Hu, Y. Zhai, C. Zhu, X. Lu, Dual-Site Trigger Electronic Communication Effects to Accelerate H_2_O_2_ Activation for Colorimetric Sensing of Uranyl Ions in Seawater, Adv. Funct. Mater., 34 (2024) 2406380.

[9] R. Zeng, Q. Zhong, R. Zhao, M. Qiu, Y. Peng, H. Guo, C. Sun, B. Zhang, X. Yan, Y. Wang, S. Guo, Precise Strain Tuning of PtPdRhNi Nanozyme Boosts Multi-Pathogen and Multi-Model Antibacterial Therapy, Adv. Mater., 38 (2026) e18526.

[10] Z. Yu, Z. Xu, R. Zeng, M. Xu, M. Zou, D. Huang, Z. Weng, D. Tang, Tailored Metal-Organic Framework-Based Nanozymes for Enhanced Enzyme-Like Catalysis, Angew. Chem. Int. Ed., 64 (2025) e202420200.

[11] Z. Yu, M. Xu, M. Xu, J. Gu, H. Xue, D. Tang, Fe–N_3_S Single-Atom Nanozyme with Asymmetric Coordination for Ultra-Low-Background Colorimetric Immunoassays, Anal. Chem., 98 (2026) 1783-1792.

[12] W. Liu, Q. Chen, J. Wu, F. Zhang, L. Han, J. Liu, H. Zhang, Z. Hao, E. Shi, Y. Sun, R. Zhang, Y. Wang, L. Zhang, Asymmetric Coordination of Iron Single-Atom Nanozymes with Efficient Self-Cascade Catalysis for Ferroptosis Therapy, Adv. Funct. Mater., 34 (2024) 2312308.
